# Association of ABO blood groups and Rh Factor with autism spectrum disorder, down syndrome, and attention deficit hyperactivity disorder among children in the Saudi population

**DOI:** 10.12669/pjms.42.6.13620

**Published:** 2026-06

**Authors:** Thamir Al-Khlaiwi, Sultan Ayoub Meo, Manan Alhakbany, Aurangzeb Taj Halepota, Muhammad Iqbal

**Affiliations:** 1 Thamir Al-Khlaiwi, Department of Physiology, College of Medicine, King Saud University, Riyadh, Saudi Arabia; 2 Sultan Ayoub Meo, Department of Physiology, College of Medicine, King Saud University, Riyadh, Saudi Arabia; 3 Manan Alhakbany, Department of Physiology, College of Medicine, King Saud University, Riyadh, Saudi Arabia; 4 Aurangzeb Taj Halepota, Department of Physiology, College of Medicine, King Saud University, Riyadh, Saudi Arabia; 5 Muhammad Iqbal, Department of Physiology, College of Medicine, King Saud University, Riyadh, Saudi Arabia

**Keywords:** Autism spectrum disorder, Attention Deficit Hyperactivity Disorder, Blood group, Down syndrome, Neurodevelopmental disorders, Rh factor

## Abstract

**Objectives:**

This study aimed to investigate the association between ABO blood groups and Rh factors with Autism Spectrum Disorder, Down Syndrome, and Attention Deficit Hyperactivity Disorder among children in the Saudi population.

**Methodology:**

In this retrospective observational study, data on 269 children (200 male and 69 female) diagnosed with Autism Spectrum Disorder, Down syndrome, and Attention Deficit Hyperactivity Disorder were obtained from the electronic medical records system of King Khalid University Hospital (KKUH), Riyadh, Saudi Arabia between 2015 and 2024. The dataset includes age, gender, blood group type, and Rh factor for children diagnosed with Autism Spectrum Disorder, Down syndrome, and Attention Deficit Hyperactivity Disorder, as well as information on their parents.

**Results:**

This study comprises 200 male children (74.3%) and 69 female children (25.7%). The most common blood group among all groups was O+ (Autism Spectrum Disorder: 40%, Down syndrome: 31.4%, Attention Deficit Hyperactivity Disorder 39.4%), followed by A+ and B+. Rh-negative types were rare, and the AB+ blood group was underrepresented across the study population. Similarly, the mother’s blood group type did not significantly differ across diagnoses, χ^2^(12) = 17.24, p = 0.141. Blood group O+ was the most common maternal (43.5%), and paternal (36.4%) blood type. The mother’s and father’s blood groups showed no statistically significant association with the child’s neurodevelopmental disease diagnosis.

**Conclusions:**

Our findings suggest no association between blood groups and Autism Spectrum Disorder, Down syndrome, and Attention Deficit Hyperactivity Disorder among children and their healthy parents in the Saudi population. The most common blood group of children and mothers was O+. However, blood groups O+ and A+ were the most prevalent blood groups among fathers.

## INTRODUCTION

The Autism spectrum disorder (ASD), Down syndrome (DS), and attention deficit hyperactivity disorder (ADHD) are multifactorial neurodevelopmental conditions that markedly impair brain biology and socio-behavioural functioning. The diseased children with ASD, DS, and ADHD and their families are facing multiple challenges in their daily lives, due to neurodevelopmental and neuropsychiatric problems, in addition to intellectual disability.^1^ This triad of disorders, ASD, DS and ADHD, is diagnosed with co-existing neurological conditions.^2^ However, the correlation has not been fully defined with numerous environmental and genetic factors.

The occurrence of these disorders, ASD, DS, and ADHD, is steadily increasing worldwide. The incidence of ASD is approximately one in 31 (3.2%) children in the age group less than 10 years. ASD occurs in various racial, ethnic, and socioeconomic groups, and is over three times more common among boys than girls.^3^ The prevalence is variable in multiple states: ASD (3.11%) and ADHD (9.50%). The “disparities persisted across sociodemographic subgroups, with variations in prevalence rates.”^4^ However, the occurrence of DS, or chromosome 21 trisomy, has an incidence of 1/1000-1/1100 newborns and is a significant cause of intellectual disability.^5^ Worldwide, about 1.81 million people have DS.^6^

The scientific literature highlights the latent role of genetic, environmental, and immunological factors in neurodevelopmental disorders. Worldwide, significant efforts have been made to understand the roles of genetic and environmental contributors.^7^,^8^ However, the relationship between blood groups and Rh factors remains unclear, especially in various populations. The “blood groups are polymorphic, antigenic, genetic substances established on the surface of Red Blood Cells (RBCs) and other cells and tissues. In 1900, Karl Landsteiner discovered the ABO blood group. ABO and Rhesus blood types are the major human blood type systems with principal importance in transfusion medicine”.^9^

The ABO blood type system encompasses four major “ABO phenotypes: A, B, O, and AB.” The ABO blood group system is associated with several diseases, including gastric and duodenal ulcers,^10^ Type-2 diabetes mellitus,^11^ anxiety,^12^ and cancers.^13^-^14^ The limited literature established the liaison between “ABO blood group” and the neurodevelopmental disorders, ASD, DS, and ADHD among specific ethnic groups. Due to ethnicity differences and their impact on the prevalence of the diseases, the present study aimed to explore the association between ABO blood groups and Rh factors among children diagnosed with ASD, DS, and ADHD and their healthy parents in the Saudi population.

## METHODOLOGY

This retrospective observational study was conducted in the “Department of Physiology, College of Medicine, King Saud University, Riyadh, Saudi Arabia.”

### Data collection:

After obtaining ethical approval, the two members of the research team recorded the data. The data were retrieved from the electronic system and the medical record unit at the Pediatric Clinic of King Khalid University Hospital (KKUH) between 2015 and 2024. The inclusion criteria were all patients who were diagnosed with “Autism Spectrum Disorder (ASD), Down syndrome (DS), and Attention Deficit Hyperactivity Disorder (ADHD),” attended King Khalid University Hospital (KKUH), Pediatric clinic and residing in Saudi Arabia. At the same time, the exclusion criteria were patients who were not under the above-mentioned developmental diseases or who were not living in Saudi Arabia. After retrieving the data for all patients who met the inclusion criteria, the blood groups of the mother, father, and child were also recorded. We collected 269 participants’ data.

### Ethics statement:

This study was approved by the Institutional Review Board, College of Medicine, King Saud University, Riyadh, Saudi Arabia (No. E-24-8717; dated April 23, 2024).

### Statistical analysis:

The frequencies and percentages were used to describe the categorically measured variables. The Bivariate chi-squared test was used to determine the association between the children’s diagnoses, their blood types, and parental blood types. However, the Likelihood ratio-corrected chi-squared test was used where needed for contingency tables that violated the chi-squared test assumptions, such as having significantly fewer than expected counts in specific cells. The IBM SPSS statistical computing program, Version 30, was used for analysis. Alpha significance level was considered at the <=0.050 level.

## RESULTS

The study involved a sample of 269 children diagnosed with ASD, DS, or ADHD. The gender distribution indicated a predominance of male children (n=200, 74.3%) over female children (n=69, 25.7%). The mean age of the children was 12.56 years (SD = 5.98), with most participants falling within the 10-17-year age range (43.9%), followed by children aged 5-9 years (34.2%), ≥18 years (15.6%), and 2-4 years (6.3%). In terms of nationality, the vast majority of children were Saudi nationals (91.1%), while only 8.9% were non-Saudi. The average maternal age was 31.89 years (SD = 6.52), and the average paternal age was 37.83 years (SD = 8.51), reflecting typical reproductive age ranges in the region. Geographic distribution showed that most families resided in the central provinces of Saudi Arabia (69.5%), followed by the western provinces (20.8%), with only minor representation from the eastern (4.5%), northern (3%), and southern provinces (2.2%).

The distribution of medical diagnoses, family history, and blood group types among children and their parents within the study sample (N = 269) are presented in Table-II. Among the participating children, the majority were diagnosed with ASD, comprising 68.8% of the sample (n=185), followed by DS (19%, n = 51), and ADHD (12.3%, n = 33). These distributions reflect the primary focus of the study on neurodevelopmental disorders, particularly ASD, which was most prevalent in the sample (Table-II). Among the children, the most common blood groups were O+ (38.3%) and A+ (35.3%), followed by B+ (18.2%). Rh-negative blood groups were relatively rare: A− (1.9%), B− (1.9%), O− (3%), and AB+ (1.5%). Notably, the O+ blood group was also most common among mothers (43.5%) and fathers (36.4%), followed by A+ (mothers: 29%; fathers: 33.5%) and B+ (mothers: 18.2%; fathers: 19.3%). These distributions suggest a shared familial pattern, with Rh-negative types being consistently uncommon in both parents and children Table-II).

**Table-I:** Sociodemographic characteristics of the study participants (N=269).

Demographic variables	Frequency	Percentage
***Gender of the child***		
Male	200	74.3
Female	69	25.7
***Age***		
2-4 years	17	6.3
5-9 years	92	34.2
10-17 years	118	43.9
≥18 years	42	15.6
***Nationality***		
Saudi	245	91.1
Non-Saudi	24	8.9

**Table-II:** Characteristics of study participants, children, and their parents (N=269).

Characteristics	Frequency	Percentage
***Diagnosis of the child (Neurodevelopmental Disorder)***		
ASD	185	68.8
Down Syndrome	51	19
ADHD	33	12.3
***Children’s blood group type***		
A+Ve	95	35.3
A-Ve	5	1.9
B+VE	49	18.2
B-Ve	5	1.9
O+Ve	103	38.3
O-Ve	8	3
AB+Ve	4	1.5
***Healthy mother’s blood group type***		
A+Ve	78	29
A-Ve	8	3
B+VE	49	18.2
B-Ve	4	1.5
O+Ve	117	43.5
O-Ve	8	3
AB+Ve	5	1.9
***Healthy father’s blood group type***		
A+Ve	90	33.5
A-Ve	3	1.1
B+VE	52	19.3
B-Ve	6	2.2
O+Ve	98	36.4
O-Ve	11	4.1
AB+Ve	9	3.3

### Bivariate Associations Between ABO Blood Groups and Child Diagnosis:

Table-III explores medical-related characteristics of children and their healthy parents about the child’s principal diagnosis of ASD, DS and ADHD. The most common blood group among all groups was O+ (ASD: 40%, Down syndrome: 31.4%, ADHD: 39.4%), followed by A+ and B+. Rh-negative types were rare, and AB+ blood group representation was minimal across all diagnoses. Similarly, the healthy mother’s blood group type did not significantly differ across diagnoses, χ^2^(12) = 17.24, p = 0.141. Again, O+ was the most frequent maternal blood type with their children (ASD: 43.8%, Down syndrome: 35.3%, ADHD: 54.5%). Other blood types appeared in minor frequencies, and no distinct pattern emerged to suggest diagnostic clustering. The healthy father’s blood group type also showed no statistically significant relationship with child diagnosis, χ^2^(12) = 12.98, p = .371. O+ and A+ were the most prevalent blood groups among fathers, with similar distributions across all three diagnostic categories. Furthermore, analysing blood group types, when dummy-coded and when aggregated into major blood types regardless of the Rh factor (A group, B group, O Group, and AB group), showed no statistically significant associations with those disease types among offspring.

**Table-III:** Blood groups of children and their parents.

Blood groups of children and their parents	Children with the principal diagnosis				
Blood groups	ASD n=185	DS n=51	ADHD n=33	Test Statistics	p-value
***Children’s blood group type***					
A+Ve	60 (32.4)	22 (43.1)	13 (39.4)	χ^2^(12)=13.17	0.357
A-Ve	2 (1.1)	1 (3.9)	1 (3)		
B+VE	35 (18.9)	8 (15.7)	6 (18.2)		
B-Ve	5 (2.7)	0	0		
O+Ve	74 (40)	16 (31.4)	13 (39.4)		
O-Ve	7 (3.8)	1 (2)	0		
AB+Ve	2 (1.1)	2 (3.9)	0		
***Healthy mother’s blood group type***					
A+Ve	50 (27)	18 (35.3)	10 (30.3)	χ^2^(12)=17.24	0.141
A-Ve	3 (1.6)	3 (5.9)	2 (6.1)		
B+VE	37 (20)	10 (19.6)	2 (6.1)		
B-Ve	3 (1.6)	1 (2)	0		
O+Ve	81 (43.8)	18 (35.3)	18 (54.5)		
O-Ve	8 (4.3)	0	0		
AB+Ve	3 (1.6)	1 (2)	1 (3)		
***Healthy father’s blood group type***					
A+Ve	54 (29.2)	23 (45.1)	13 (39.4)	χ^2^(12)=12.98	0.371
A-Ve	1 (0.5)	1 (2)	1 (3)		
B+VE	40 (21.6)	8 (15.7)	4 (12.1)		
B-Ve	3 (1.6)	1 (2)	2 (6.1)		
O+Ve	71 (38.4)	15 (29.4)	12 (36.4)		
O-Ve	9 (4.9)	2 (3.9)	0		
AB+Ve	7 (3.8)	1 (2)	1 (3)		

## DISCUSSION

The present study results revealed that the predominance of neurodevelopmental disorders is more frequent among male children than females. ASD was the most common disorder, followed by DS and ADHD. There was no association between blood groups and Autism Spectrum Disorder, Down syndrome, and Attention Deficit Hyperactivity Disorder among diagnosed children and their healthy parents.

Understanding blood groups and Rh factors among children with neurodevelopmental disorders across populations is crucial. This understanding is particularly vital for the specific Saudi population we studied. The recent research, Belali et al.^15^, reported that the most prevalent ABO blood group among the Saudi population, it was noted that the blood group O+ was the most prevalent blood type (38.68%), followed by types A+ (24.81%), B+ (20.90%), O- (7.22%), AB+ (4.72%), A- (2.59%), B- (0.81%) and AB- (0.23%). Similarly, in another study, Alanazi et al. (2022)^16^ reported that “blood group O was the most prevalent (50.1%), followed by B (32.0%), A (14.4%), and AB (3.6%). RhD positivity was relatively high (93.3%)” in the Saudi population.

Al-Salihy et al.^17^ investigated the relationships between ABO blood groups, Rh factors, and ASD in Iraq. The authors found that blood group O+ve was the most prevalent. Interestingly, the authors identified that the AB+ blood group was associated with a significantly lower risk of ASD and may have a protective function. Similarly, in the present study, we found that the most common blood group among all groups was O+ for ASD, and the AB+ve blood group had a lower risk of ASD. The authors also explored the blood group distribution among mothers and fathers of children with ASD. Neither maternal nor paternal blood group showed a link. This demonstrates no relationship between parental blood group and ASD risk in children. Similarly, we did not find any link between the parental blood group and ASD, DS and ADHD.

The study conducted by Wu et al. (2012)^18^ found no relationship between blood groups and neurodevelopmental risk factors. Ravi et al. and Mendonca et al. (2024)^19^ investigated the link between blood group and ASD risk. The authors identified a link between Rh and ABO blood group incompatibilities, which can increase the risk of neurodevelopmental risks. Moreover, Ajdacic-Gross V et al.(2025)^20^ identified an association between the AB blood group and an increased risk of neurodevelopmental disorders. However, this study did not specifically focus on ASD, DS, and ADHD.

The recent literature has explored the association between blood group antigens and the risk of neurodevelopmental disorders. The AB + blood group is associated with a significantly lower risk of ASD, suggesting a novel protective factor. No significant associations were observed between other blood groups and Rh factors and ASD risk. Additionally, there were no significant differences in blood group distributions among children and their parents with ASD, other neurodevelopmental disorders.^17^

The literature has established some relationship between blood group antigens and their contribution to inflammatory responses.^21^ It is reasonable that some blood groups could indirectly affect ASD, DS, and ADHD risk. The literature has linked Rh incompatibility between mother and fetus with an increased risk of neurodevelopmental disorders, due to hemolytic disease of the fetus and newborn and resulting bilirubin toxicity. Increased bilirubin in neonates can cause adverse neurological effects, including neurodevelopmental disorders.^22^

The literature highlights the complex pathophysiology of neurodevelopmental disorders such as ASDs,^21^ DS and ADHD. It was also hypothesized that the blood type of the parents may be a risk factor for ASD with unknown genetic mechanisms. While the association of blood group and certain diseases was observed^11^,^23^, however, no association was discovered between the ABO blood type of parents and children with ASD.^18^

### Strengths of the study:

This study is based on a sufficient sample size of 269 individuals with ASD, DS, and ADHD from the Saudi Arabia population. The diagnosis was established entirely through the university hospital’s electronic database, providing a standardized diagnostic setting. The data were obtained from the university hospital’s electronic databases, which are exceptionally reliable sources.

### Limitations:

Some confounding factors may have influenced the results, including regional variations, environmental, genetic, or sociocultural factors. These factors may affect the distribution of ASD, DS, and ADHD cases in the country.

## CONCLUSIONS

Our findings suggest no association between blood groups and Autism Spectrum Disorder, Down syndrome, and Attention Deficit Hyperactivity Disorder among children. Autism Spectrum Disorder, Down syndrome, and Attention Deficit Hyperactivity Disorder were more frequent among male than female children. Autism Spectrum Disorder was the most common disorder, followed by Down syndrome and attention deficit hyperactivity disorder.

### Author`s Contributions:

**TAK, SAM,** study concept, supervision, obtaining a grant, writing the manuscript.

**MA, ATH, MI,** literature review, data collection, checking, entry, and analysis.

**TAK:** Responsible and accountable for the accuracy or integrity of the work.

All authors haves read and approved the final version of the manuscript.
